# Exploring oral antibiotics for antibiotic stewardship in nonoperative management of complicated appendicitis in pediatric patients

**DOI:** 10.1017/ash.2025.74

**Published:** 2025-04-25

**Authors:** Rebecca John, Alvaro E. Galvis, Robert F.T. Bucayu, John Schomberg, Yigit Guner, Antonio Arrieta, Delma Nieves

**Affiliations:** 1 Division of Surgery, CHOC Children’s Hospital, Orange, CA, USA; 2 Division of Infectious Diseases, CHOC Children’s Hospital, Orange, CA, USA; 3 Department of Pediatrics, University of California, Irvine, CA, USA; 4 School of Medicine, University of California, Irvine, CA, USA; 5 Department of Trauma/Nursing Administration, CHOC Children’s Hospital, Orange, CA, USA

## Abstract

**Introduction:**

Culture data may help determine antibiotic administration options for nonoperative complicated appendicitis. Variability exists in treatment strategies, from solely using intravenous therapy, including outpatient parenteral antibiotic treatment (OPAT), to transitioning to oral (PO) antibiotics. We hypothesize that most patients have an oral antibiotic option based on culture results and that there is no increased rate of readmission due to treatment failure with oral antibiotics.

**Methods:**

This was a single-center retrospective cohort study reviewing antibiotic treatment of pediatric patients treated with nonoperative management for complicated appendicitis with abscesses percutaneously drained by Interventional Radiology (IR). We compared case-mix demographic variables, choice and route of antibiotic therapy, culture data, and clinical outcomes between those who exclusively received parenteral antibiotics therapy (PAT) and those who were switched to oral therapy (PO).

**Results:**

We identified 54 cases of nonoperative complicated appendicitis who underwent IR abscess drainage from 2014 to 2019. Forty-five [83%] patients completed therapy with PAT and 9 with PO; forty-six of 54 patients (85%) patients had an oral antibiotic(s) option based on sensitivities. Readmissions and complications included 6 (11%) patients. Three (50%) patients were readmitted due to antibiotic treatment failure with worsening of abscess formation, 2 due to PICC (peripherally inserted central catheters) issues, and 1 due to a drug reaction.

**Conclusions:**

Most patients with nonoperative complicated appendicitis can be transitioned to oral antibiotic options based on the culture susceptibility profiles.

Appendicitis is the most common cause of abdominal pain requiring emergent surgical intervention in pediatrics yet there exists significant variability in its antibiotic management.^1^ Complicated appendicitis includes those with either a grossly identifiable or microscopic perforation in the appendix, with a phlegmon, pus, or abscess in the abdomen.^2^ Complicated appendicitis occurs in 30%–60% of appendicitis cases, and this is especially true in younger patients.^3^ Cases with extended duration of symptoms or with imaging revealing extensive inflammation, particularly to the base of the appendix and cecum are often more complex to manage. Initial nonoperative treatment of complicated appendicitis includes administration of antibiotics and percutaneous drainage by an interventional radiologist (IR) if there is an accessible intra-abdominal abscess.^4^ Although precise definitions and management of complicated appendicitis may vary between surgeons and hospitals, it is the standard of care to treat those children with ongoing antibiotic therapy.^5,6^

Antimicrobials are among the most used medications within the pediatric population, yet considerable variability in choice and route of administration exists for complicated appendicitis. Overuse and inappropriate use of antibiotics contribute to rising worldwide antibiotic resistance and so antimicrobial stewardship is imperative.^7^ Peripherally inserted central catheters (PICCs) are commonly placed in children with ongoing parenteral antibiotics in the medical management of appendicitis. However, there are disadvantages to PICCs, including painful insertion, activity restrictions, deep venous thrombosis, and risk of mechanical and infectious complications.^8–10^ As of yet, there is no consensus on whether it is best practice to complete the course of antibiotics parenterally, including the use of outpatient parenteral antibiotic treatment (OPAT) via a PICC or transition to oral (PO) antibiotics in these complex patients.^11^ In patients with operative appendicitis studies have delineated certain criteria to be met to safely transition from IV to oral antibiotics including using culture data if available as well as oral tolerance, remaining afebrile for 24 hours, and improvement in pain.^12,13^ However, there is paucity of data for patients managed nonoperatively. Therefore, many clinicians rely on extrapolation of operative studies into nonoperative patients to determine a treatment course.

In this retrospective study, the primary outcome studied was availability of an oral antibiotic option based on culture and susceptibility results for patients undergoing nonoperative management for complicated appendicitis after IR drainage. Secondary outcomes included readmission rate due to clinical failure (relapse of initial signs/symptoms), PICC complications (catheter-related bloodstream infections or mechanical failures including clotting and dislodgment), length of hospital stay, time to IR drainage (as this plays a role in accuracy of culture results), and whether lab values (CBC and inflammatory markers) differed according to route of antibiotic therapy chosen.

## Methods

This was a single-center study reviewing hospitalized pediatric patients (age up to 18 yr) with complicated appendicitis treated nonoperatively, who underwent percutaneous drainage and had available culture results from January 1st, 2014, through December 31st, 2019. The study was approved by the local institutional review board at Children’s Hospital of Orange County (CHOC), IRB # 200215. Study data were collected and managed using REDCap (Research Electronic Data Capture) electronic data capture tools hosted at CHOC.^14,15^

We collected data on clinical presentation, diagnostic studies obtained, including laboratory and imaging studies, microbiologic culture results, and timing of IR drainage. Few patients underwent more than one drainage but did not require antibiotic adjustments based on culture results. The antibiotic choice and route while hospitalized and at discharge, as well as the total duration of antibiotics, were recorded. We reviewed outpatient clinic visit records and collected follow-up data. Any complications after discharge including readmissions were identified. Patients who had a diagnosis of complicated appendicitis but underwent immediate surgical management, or who were not a candidate for percutaneous drainage or whose cultures did not yield microbial data were excluded from the study. All patients with simple appendicitis were excluded. The attending surgeon determined if the patient should undergo nonoperative management or was an operative candidate.

Patients were identified based on ICD9 codes using the inclusion criteria. Two groups were established according to the route of antibiotics administered to complete therapy; patients who completed their antibiotic course with parenteral therapy (PAT) were compared to patients who were transitioned to oral antibiotic therapy (PO). We then reviewed and compared the data between these two groups.

Frequencies and proportions of patients were reported for all categorical characteristics stratified by parenteral and oral antibiotic use status. Continuous variables were reported using mean, median and standard deviation. A Wilcoxon rank-sum test was used to detect statistically significant differences in distribution of continuous variables among groups receiving parenteral and oral antibiotics. A Fisher’s exact test was used to detect significant differences in proportions in categorical variables when stratifying by antibiotic status. Statistical tests were limited by power but were carried out to detect large effects that could be contributed to confounding of clinical case mix, or a large effect associated with difference in treatment. Statistical significance was defined with a *p* value <0.05. Statistical analysis comparing the PAT and PO groups was accomplished using R Statistical Programming Language Version 4.3.^16^

## Results

### Demographics and clinical outcomes

From January 1^st^, 2014, to December 31^st^, 2019, 54 patients at our institution underwent initial nonoperative management for complicated appendicitis with cultures obtained by IR percutaneous drainage. Forty-five patients (83%) finished treatment with PAT including 44 (81%) who were discharged to complete outpatient treatment with a PICC (OPAT), and 1 (2%) who completed antibiotic treatment inpatient with IV antibiotics. Nine patients (16%) were transitioned to oral antibiotics to complete their treatment and were in the PO group. Patients were 59% males, 56% identified as Hispanic, and 52% were between the ages of 5 and 12 years old; the mean age for the PAT group was 9.8 years and for the PO group was 10.2 years; Table 1 shows this demographic data.

**Table 1.** Descriptive analysis of cases stratified by antibiotics route at completion of therapy

Patient Characteristic	PAT n = 45	PO n = 9	p-value
**Age** (Mean, Median, Standard Deviation)	9.9,11,4.2	9.8,10,5.1	0.85
**Age Group**			0.31
Infant <2 yr	2 (4.4%)	0 (0%)	
Young children 2–5 yr	5 (11.1%)	3 (33.3%)	
School Age 5–12 yr	25 (55.5%)	3 (33.3%)	
Adolescent > 12 yr	13 (28.8%)	3 (33.3%)	
**Gender**			0.06
Female	21 (46.6%)	1 (11.1%)	
Male	24 (53.3%)	8 (88.8%)	
**Race**			0.4
African American	0 (0%)	1 (11.1%)	
Asian	4 (8.8%)	1 (11.1%)	
Other	9 (20.0%)	1 (11.1%)	
White	31 (68.8%)	6 (66.6%)	
Not Stated	0(0%)	0 (0%)	
**Ethnicity**			1
Hispanic	25 (55.5%)	5 (55.5%)	
Not Hispanic	20 (44.4%)	4 (44.4%)	
**Is There a PO Option**			
No	8 (17.8%)	0	
Yes	37 (82.2%)	9	
**Total Days Inpatient (mean, median, standard deviation)**	6.9,5,5.2	8.3,9,3.9	0.08
**Readmission/re-evaluation due to complications**			0.25
No	41 (93.2%)	7 (77.8%)	
Yes	4 (8.8%)	2 (22.2%)	
**Complications from PICC Line**			
No	44 (97.8%)	8 (88.9%)	
Yes	1 (2.2%)	1* (11.1%)	

*One PO patient was readmitted with arm pain and found to have clot at PRIOR picc site (removed prior to dc).

**Figure 1.**
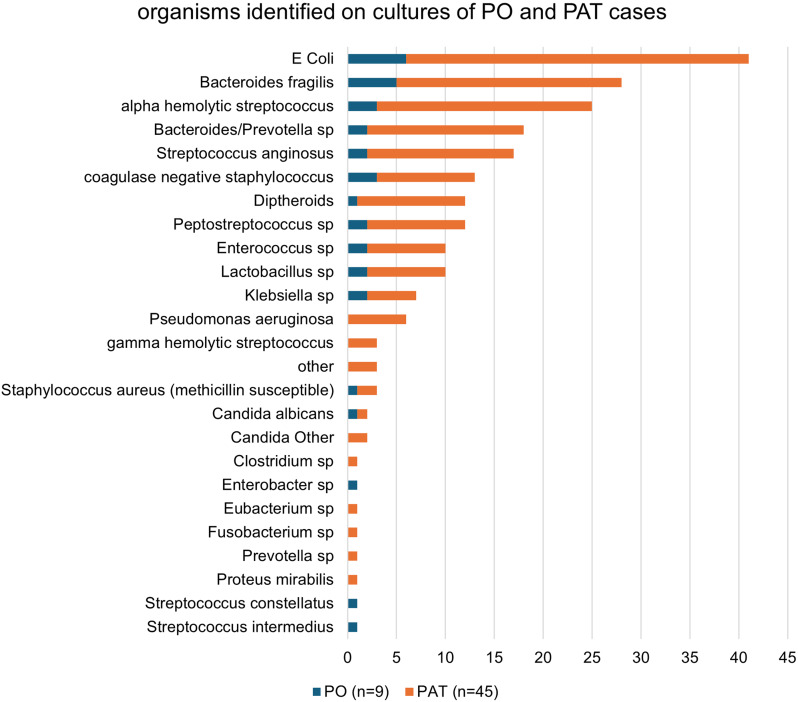
Distribution of culture results.

Based on the identified organism and available susceptibilities, 46 of 54 patients (85%) which includes 37 (82.2%) PAT patients, and all 9 PO patients had an oral antibiotic(s) as a treatment option. There were 8 PAT patients whose culture results did not include susceptibilities, however based on typical organism characteristics (anaerobes) an additional 5 would have been appropriately treated with PO antibiotics for a total of 94% that could have an oral antibiotic option. The three most common organisms found in PAT patients were *E.* coli in 35 (77.7%), *Bacteroides fragilis* in 23 (51.1%) and alpha-hemolytic streptococcus in 22 (48.8%). Of the *E. coli* isolates 91.4% (39/41) had a PO option with the remaining 2 isolates being extended-spectrum beta-lactamase (ESBL) strains. Other notable organisms included all *Pseudomonas aeruginosa* (*P. aeruginosa*) that were susceptible to quinolones as a treatment option. Figure 1 shows the organisms identified in all cases, distinguished as PO vs PAT.

Overall, 6 (11%) patients were readmitted, 4 (7%) on PAT and 2 (4%) in the PO group (p-value = 0.25). Of the 4 PAT patients who required readmission, 2 (50%) were due to antibiotic treatment failure with worsening of abscess formation. One PAT patient developed an acute generalized exanthematous pustulosis reaction while on piperacillin-tazobactam, and one patient had a PICC line issue. Of the 2 PO patients readmitted, one was a treatment failure and the other had a clot at a prior PICC site (PICC was removed before discharge). All 3 patients who came back as treatment failure required further IR drainage procedures or surgical management and all had *Escherichia coli (E. coli)* and a mix of some anaerobes in their initial culture.

Mean hospital length of stay (LOS) was 6.9 days (median of 5 days) for those patients who completed therapy on parenteral antibiotics. For the 9 patients who completed their regimen with PO antibiotics, the LOS was 8.3 days (median 9 d). The time from admission to IR drainage was within 24 hours in 40 [74.1%], within 24–48 hours in 4 [7.41%], within 48-72 in 2 [3.7%] and done after 72 hours in the remaining 8 [14.8%] and did not differ between groups. Laboratory values (WBC, hemoglobin, platelets, differential and inflammatory markers) were recorded at admission, at hospital discharge and at end of therapy (Table 2) but also no statistical differences were noted between groups. Mean total antibiotic duration was 18.7 days (median 18, SD = 5.4) for the PAT group and 20 (median 16, SD = 9.3) days for the PO group (*P* = 0.45). The shortest oral antibiotic course following an intravenous course was no less than 5 days. Upon admission, the most common antibiotic regimen was ceftriaxone and metronidazole (79.6%) and the most common antibiotic used at discharge for OPAT was ertapenem (64.4%) followed by piperacillin-tazobactam (20%). Most used antibiotics on discharge for the PO group were metronidazole (44.4%), amoxicillin/clavulanate (33.3%), levofloxacin (33.3%), and ciprofloxacin (22.2%), often used in combinations.

**Table 2.** Distribution of lab results stratified by time point and antibiotic treatment status

	PAT		PO		
		p-value		p-value	p-value between groups
Hemoglobin (gm/dL)					
Admission	12.1, 11.9, 1.5	0.02	12.2, 12.7, 1.5	0.48	0.59
Discharge	11.1, 10.9, 1.3		11.5, 11.5, 1.1		0.36
EOT	11.2, 11.0, 1.37		11.9, 11.8, .53		0.37
WBC (K/uL)					
Admission	17.2, 16.3, 6.4	<0.0001	19.2, 16.1, 12.2	0.03	0.96
Discharge	9.5, 8.6, 3.1		10.1, 9.6, 3.0		0.48
EOT	7.3, 7.6, 1.6		9.0, 8.9, .36		0.01
Neutrophil (%)					
Admission	73.7, 77.8, 13.7	<0.0001	64.5, 66.7, 19.4	0.18	0.25
Discharge	55.1, 58.1, 14.6		60.7, 60, 16.8		0.45
EOT	NA		NA		NA
Bands (%)					
Admission	8.2, 5, 10.3	NA	22.3, 26, 7.2	NA	0.02
Discharge	2.2, 1.0, 2.6		2.6, 1.5, 2.9		0.64
EOT	NA		NA		NA
Platelets (K/uL)					
Admission	334.5, 308.5, 113.7	0.6	322.7, 321, 110	0.001	0.92
Discharge	449.4, 447.5, 114.5		432.3, 429.5, 84.9		0.67
EOT	436.5, 440.5, 133.6		434, 308, 221.7		0.59
CRP (mg/L)					
Admission	187.3, 174.1, 129.6	NA	159.3, 157.8, 76.7	NA	NA
Discharge	NA, 32, NA		NA, 22.6, NA		
EOT	NA, NA, NA		NA, NA, NA		
ESR (mm/hr)					
Admission	63.5, 50, 30.5	0.4	28.5, 28.5, 2.2	0.66	0.13
Discharge	63.1, 76.5, 34.8		58.5, 58.5, 4.9		0.64
EOT	30.6, 18, 28.2		11, 11, NA		0.63

*Results shown as mean, median, standard deviation.

## Discussion

Pediatric complicated nonoperative appendicitis is a common condition with significant heterogeneity in management. We identified that 82.2% of the PAT cohort had an available PO antibiotic regimen option based on the susceptibility profiles identified on culture and presumed to be up to 91.1% of PAT (94% including PO patients) since some organisms without susceptibilities performed were ones typically covered with oral antibiotics. Historically, PICC placement and OPAT are routinely used to administer antibiotics for patients with nonoperative complicated appendicitis as reported in the literature.^6,17–19^ While initial empiric choices of “broad-spectrum” antibiotics including carbapenems are also often used, previous studies have shown the combination of metronidazole and a third-generation cephalosporin to be effective in the management of complicated appendicitis.^18,19^ Our most common initial choice of empiric antibiotics was ceftriaxone and metronidazole; at the time of discharge, the most common parenteral antibiotic was ertapenem. Based on culture data in this study ESBL organisms were rare and all *P. aeruginosa* were susceptible to ciprofloxacin so the carbapenem may have been chosen for ease of administration rather than based on microbiologic indication. The frequent use of carbapenems is concerning from an antibiotic stewardship standpoint; it may lead to increase in infections due to antimicrobial-resistant bacteria which has been reported to lead to increased morbidity and mortality, longer hospital stays, and increased health care costs.^7,17,20^

Studies have shown the use of oral antibiotics after an initial course of IV antibiotics to be effective in the management of complicated operative appendicitis. Adibe et al reported a 14-day course combining initial IV antibiotics followed by oral metronidazole and trimethoprim/sulfamethoxazole to be as safe as and more cost-effective than a 14-day course of IV antibiotics.^21^ Similarly, a randomized control trial of 150 pediatric patients with operative complicated appendicitis by Frasser et al demonstrated no difference between a full IV antibiotic course group or an IV plus PO group in the time to full oral intake, length of postoperative hospitalization, total health care visits, or postoperative abscess rate.^5^ Other studies have demonstrated similar results.^22–24^ Of note microbiological data were not utilized in these prior studies to guide decisions on oral regimen as intraoperative cultures were not sent.

We found readmissions in both groups but were in such small numbers no clinical significance was detected (*P* = 0.25). Interestingly, PO patients had longer LOS compared to PAT (*P* = 0.02), and on average PO patients were on antibiotics longer, but we could not detect significant differences between the groups. Some patients were observed on oral antibiotics for 24–48 hours to ensure they continued to improve off IV antibiotics, increasing LOS. With the small size of our PO cases, a true correlation with length of stay cannot be made. Additionally, no differences were seen in CBC and inflammatory markers between groups, but this could also be related to small sample size.

Our data suggests the use of cultures obtained at the time of IR drainage when done early after presentation of nonoperative complicated appendicitis may be helpful to guide appropriate PO regimen choice and help prevent the use of unnecessary broad-spectrum PAT. Most patients underwent IR drainage of their periappendiceal abscess within 24 hours of admission. We believe this allows for reliable culture information which then can be used to guide antibiotic management. Pediatric patients with initial nonoperative management for complicated appendicitis could benefit from waiting for culture results before considering PICC line placement and OPAT. Controversy exists on the value of obtaining cultures in patients with intra-abdominal infections, yet we believe that in a time of increasing resistance, cultures may add valuable information. Hence, obtaining cultures and susceptibilities should be considered. These data could facilitate transition to oral antibiotics significantly reducing PICC line utilization, therefore reducing healthcare costs and complications associated with OPAT.

We did not measure resistance development, nor could we assess differences in duration and cost between our OPAT and PO group as most of our cases received OPAT, however, data comparing healthcare expense found that appendicitis patients on OPAT cost an average of $67 per day versus those on oral antibiotics cost $7 per day.^11,25^ We did not collect cost data and our study was likely too small to make an actual cost comparison.

There are few studies discussing pediatric patients with complicated appendicitis who undergo nonoperative management and percutaneous abscess drainage. Most data discussing appendicitis covers simple acute appendicitis or surgical management of complicated appendicitis. This being one of the few studies covering this topic, we thought it was pertinent to present the findings, however we do acknowledge our limitations. First, this is a retrospective study in which the decision behind treatment with oral vs parenteral antibiotics is difficult to evaluate. It is possible the patients sent home on OPAT were those with more severe clinical presentation or if providers were influenced by practical reasons such as oral antibiotics palatability. In this 6-year period with a total of 54 patients, we noted a large portion (94%) had a PO option based on microbiologic data. The small number of patients who went home on PO limited our ability to perform meaningful statistical analysis regarding readmission rate and treatment failure. Our study is single center and hence may not be representative of all pediatric patients, local resistance patterns, or the current practices of other centers for nonoperative management of complicated appendicitis. We were unable to determine if any of our patients were readmitted to other centers. Our sample size overall was small limiting our ability to identify low-frequency complications of antibiotic regimens such as allergic reactions or side effects of antibiotics chosen (marrow suppression, renal or hepatobiliary toxicity). Nonetheless, given the limited literature and the treatment heterogeneity that exists, our study aimed at helping with standardization of a management approach namely utilizing cultures results obtained early in presentation to aid in choosing antibiotic regimen. A prospective randomized controlled study would be the next step to better answer this question.

In conclusion, most patients treated with PAT had an available oral antibiotic regimen based on culture and susceptibility alone. Differences in length of hospital stay, duration of antibiotics and rate of readmission were not found but our study was not well powered due to small number of PO cases. Our study adds to the body of literature supporting that in nonoperative management of complicated appendicitis in children, using culture data can help guide if transition to PO antibiotics is appropriate. The use of cultures and susceptibilities in the care of nonoperative complicated appendicitis should help reduce overutilization of broad-spectrum antibiotics and OPAT which may have a positive impact on emergence of resistance, health care costs and complications associated with use of central catheters.

## Data Availability

Any data used to produce the results reported within this study will be made available upon request to editors and reviewers. Any previously unreported custom computer code or algorithm used to generate results that are reported in the paper and central to its main claims can also be made available upon request from the corresponding author.

## Previous presentation

An initial/preliminary version of this manuscript with partial data was presented at ID week virtual conference. September 29-October 3, 2021 under the title: Antibiotic Stewardship in Nonoperative Management of Perforated Appendicitis: Oral Antibiotics Are an Alternative.

## Abbreviations:

IR: Interventional Radiology

PAT: parenteral antibiotic treatment

OPAT: outpatient parenteral antibiotic treatment

PICC: Peripherally inserted central catheters

PO: Oral antibiotics

REDCap: Research Electronic Data Capture

WBC: white blood cell count
